# A generative flow-based model for volumetric data augmentation in 3D deep learning for computed tomographic colonography

**DOI:** 10.1007/s11548-020-02275-z

**Published:** 2020-11-05

**Authors:** Tomoki Uemura, Janne J. Näppi, Yasuji Ryu, Chinatsu Watari, Tohru Kamiya, Hiroyuki Yoshida

**Affiliations:** 1 3D Imaging Research, Department of Radiology, Massachusetts General Hospital and Harvard Medical School, 25 New Chardon Street, Suite 400C, Boston, MA 02114 USA; 2 Department of Mechanical and Control Engineering, Kyushu Institute of Technology, 1-1 Sensui-cho, Kitakyushu, 804-8550 Japan; 3 Department of Radiology, Tonami General Hospital, 1-61 Shintomi-cho, Tonami, Toyama 939-1395 Japan

**Keywords:** Generative models, Data augmentation, Deep learning, Computer-aided detection, Virtual colonoscopy, Artificial intelligence

## Abstract

**Purpose:**

Deep learning can be used for improving the performance of computer-aided detection (CADe) in various medical imaging tasks. However, in computed tomographic (CT) colonography, the performance is limited by the relatively small size and the variety of the available training datasets. Our purpose in this study was to develop and evaluate a flow-based generative model for performing 3D data augmentation of colorectal polyps for effective training of deep learning in CADe for CT colonography.

**Methods:**

We developed a 3D-convolutional neural network (3D CNN) based on a flow-based generative model (3D Glow) for generating synthetic volumes of interest (VOIs) that has characteristics similar to those of the VOIs of its training dataset. The 3D Glow was trained to generate synthetic VOIs of polyps by use of our clinical CT colonography case collection. The evaluation was performed by use of a human observer study with three observers and by use of a CADe-based polyp classification study with a 3D DenseNet.

**Results:**

The area-under-the-curve values of the receiver operating characteristic analysis of the three observers were not statistically significantly different in distinguishing between real polyps and synthetic polyps. When trained with data augmentation by 3D Glow, the 3D DenseNet yielded a statistically significantly higher polyp classification performance than when it was trained with alternative augmentation methods.

**Conclusion:**

The 3D Glow-generated synthetic polyps are visually indistinguishable from real colorectal polyps. Their application to data augmentation can substantially improve the performance of 3D CNNs in CADe for CT colonography. Thus, 3D Glow is a promising method for improving the performance of deep learning in CADe for CT colonography.

## Introduction

Colorectal cancer (CRC) is the third most common cancer in terms of incidence and the second most common cancer in terms of mortality worldwide [1]. However, CRC can be prevented considerably by early detection and removal of its precursors, colorectal polyps [2]. Computed tomographic colonography (CTC) can be used both for detecting CRCs and for preventing CRCs by early detection of clinically significant polyps that could develop into cancers [3].

Deep learning based on convolutional neural networks (CNNs) has made it easy to obtain state-of-the-art results in various medical imaging tasks [4]. However, one of the limitations of deep learning is that the development of generalizable CNNs requires a large amount and great variety of training data [5]. In CTC, the available training datasets are relatively small and limited in numbers and variations.

The two principal approaches for addressing the issue of small training datasets in deep learning have included (1) transfer learning of CNNs and (2) data augmentation of training datasets. With the first approach, transfer learning, one starts with a CNN that has been pre-trained with a large number of images. The CNN is then adapted to the desired application by continuation of the training with the available domain-specific data. In CTC, transfer learning has been used successfully in training of CNNs for interpreting virtual endoluminal views [6], for detecting contrast-coated serrated polyps [7] and for performing electronic cleansing [8]. However, transfer learning has the limitations that most of the publicly available pre-trained CNNs have not been pre-trained with medical images and that most of these CNNs are 2D CNNs.

With the second approach, data augmentation, the training dataset is enhanced artificially so that the number and variety of training samples are increased. The most common method for implementing data augmentation has been the manipulation of the training samples by basic image processing operations [5]. In CTC, this approach has been used successfully for training of CNNs for detection of polyps [9] and masses [10], and for reduction in the number of false-positive (FP) detections in computer-aided detection (CADe) [11, 12]. However, the approach has the limitation that some of the image transforms that are commonly used with natural images are not appropriate for use with medical images. This can limit the number and variety of the obtainable training samples.

Recently, several studies have explored the possibility of performing data augmentation by use of generative adversarial networks (GANs) [13, 14]. In CTC, 3D GANs have been used for generation of synthetic polyps to improve the training of 3D CNNs in CADe [15]. However, the development of GANs that can generate realistic synthetic images at a high image resolution is known to suffer from various problems, such as non-convergence of the model parameters, mode collapse or training imbalance between the generator and the discriminator [5].

Flow-based generative models have several advantages over GANs [16]. Unlike GANs, they are designed to learn the distribution of the input data explicitly, thereby providing an exact latent-variable inference, log-likelihood evaluation and a meaningful latent space for performance of valid manipulations of the data. They are also highly parallelizable and can be implemented with a small memory footprint [16].

In this study, we investigated the feasibility of applying a flow-based generative model to 3D data augmentation of colorectal polyps in CTC. We hypothesized that (1) a 3D CNN that implements a state-of-the-art flow-based generative model known as *Glow* [16] (hereafter referred to as 3D Glow) can be trained to generate synthetic polyps that are visually indistinguishable from real colorectal polyps in CTC, and that (2) data augmentation by use of 3D Glow-generated synthetic polyps can be used for improving the performance of deep learning in differentiating between polyps and non-polyps in CADe for CTC. One could use successful development of 3D Glow to overcome the limitations of currently available CTC datasets, thereby enabling the training of generalizable high-performing 3D CNNs for CADe in CTC.

## Flow-based generative model

Given an observed (complicated) probability distribution, a flow-based generative model provides a bijective mapping *f* between the observed distribution and a simple, well-understood target probability distribution, such as a standard Gaussian distribution. The desired computations can then be performed on the simple target distribution rather than on the observed distribution. Such a mapping that is constructed as a sequence of invertible bijective transforms is called the *normalizing flow*. Formally, let *x* denote an observation sampled from a training dataset *D* that has an unknown probability distribution *p*(*x*). The normalizing flow can be defined as

1 $$\begin{aligned} z = f(x) = f_L {\, \circ \,} f_{L-1} {\, \circ \,} \cdots {\, \circ \,} f_1(x), \end{aligned}$$

where *z* is a latent random variable of the desired target distribution. The corresponding flow-based generative model is constructed by optimizing the parameters of the component functions of Eq. (1), $f_i$, by use of the maximum-likelihood method. The training loss is the negative log-likelihood over *D*, or

2 $$\begin{aligned} L(D) = -\frac{1}{|D|} \sum _{x {\in } D} \log p(x) \ . \end{aligned}$$

**Fig. 1:**
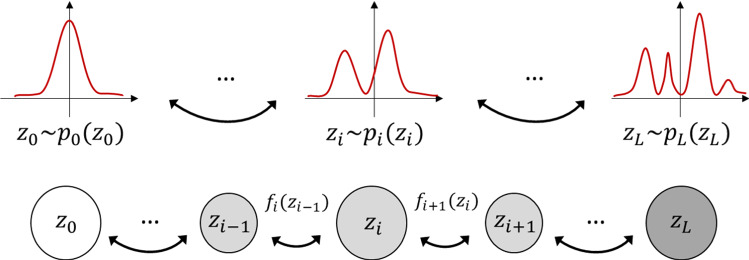
Diagram of a normalizing flow between a simple Gaussian distribution $z_0{\sim }p_{0}(z_0)$ and an observed distribution $x=z_L {\, \sim \,} p_{L}(z_L)$

Figure 1 illustrates the construction of a normalizing flow. The path traversed by the random variables, $z_i$, is a *flow*, and the full chain that is formed by the corresponding probability distributions is the normalizing flow.

## 3D Glow

**Fig. 2:**
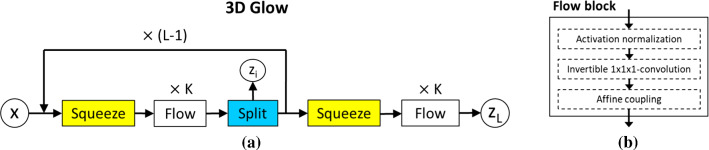
**a** Illustration of the architecture of 3D Glow. The model implements *K* levels and *L* steps of flow [16]. **b** Design of a flow block

Figure 2a illustrates the overall architecture of our 3D Glow model. The model extends the originally proposed 2D Glow framework [16] into a 3D CNN for processing of volumetric CTC images.

The model has three types of layer blocks. *Squeeze* transforms an input $w \times h \times d \times c$ tensor, where $w \times h \times d$ is the input image size and *c* is the number of channels, into a $\frac{w}{2} \times \frac{h}{2} \times \frac{d}{2} \times 8c$ tensor; thus, trading spatial size for number of channels [17]. *Flow* implements one step of the flow. *Split* splits the input tensor along the channel dimensions [16].

Figure 2b illustrates the design of a *Flow* block. There are three layers: an activation-normalization (*actnorm*) layer [16], an invertible $1 \times 1 \times 1$-convolutional layer [16] and an affine-coupling layer [18]. The *actnorm* layer performs an affine transformation of the input tensor, where the trainable per-channel scale and bias parameters are initialized to yield a mean of zero and a standard deviation of one based on the first mini-batch [16]. The invertible $1 \times 1 \times 1$-convolutional layer implements a trainable permutation that reverses the order of the input channels, where the weight matrix of the transform is initialized as a random rotation matrix [16]. The affine-coupling layer splits the input into two parts: the first *d* dimensions remain the same, whereas the latter dimensions, $d+1$ to *D*, undergo an affine transformation based on the scale and shift parameters as

3 $$\begin{aligned} y_{1:d}&= x_{1:d} \ , \end{aligned}$$

4 $$\begin{aligned} y_{d+1:D}&= x_{d+1:D} {\odot } \exp (s(x_{1:d}))+t(x_{1:d}) \ , \end{aligned}$$

where $\odot$ denotes an element-wise product and *s*(.) and *t*(.) are scale and translation functions that map ${\mathbb R}^d {\rightarrow } {\mathbb R}^{D-d}$.

It can be shown that the transformation above satisfies the basic properties required by efficient computation of the flow transformation [16]. The model is trained with the loss function of Eq. (2).

**Fig. 3:**
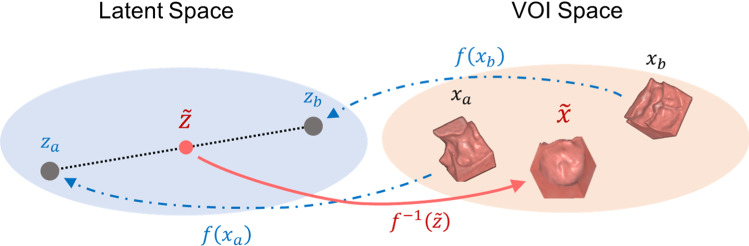
Illustration of 3D Glow-based data augmentation. Two training VOIs, $x_a$ and $x_b$, are mapped to points $z_a$ and $z_b$ of the latent space, which are used for interpolating a new point $\tilde{z}$. By mapping $\tilde{z}$ back to the VOI space, we generate a new synthetic VOI, $\tilde{x}$, which is different from, yet similar to $x_a$ and $x_b$

**Fig. 4:**
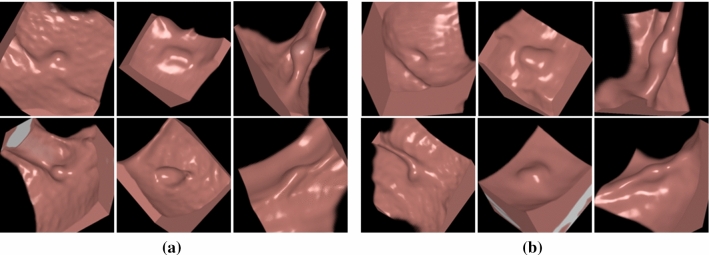
Virtual endoscopic views of the VOIs of **a** six real polyps and **b** six 3D Glow-generated synthetic polyps. Each VOI was $32\times 32\times 32$ voxels in size

## Construction of synthetic polyps

Figure 3 illustrates the principle of the 3D Glow-based data augmentation. The training of the model involves the learning of a bijective mapping from input polyp VOIs (volumes of interest, $x_a$ and $x_b$) to vectors of the latent space ($z_a$ and $z_b$) by use of the latent variable *z* and the normalizing flow of Eq. (1).

We interpolate the new samples in the latent space by use of linear interpolation between any two training samples. Given two vectors of the latent space, such as $z_a$ and $z_b$, we calculate a new sample as $\tilde{z} \sim z_a + \alpha (z_b - z_a)$, where $\alpha$ is sampled randomly from the uniform distribution and scaled linearly to $\alpha {\in }[0.4, 0.6]$ to avoid generating VOIs that would be nearly identical to the VOI of real polyps in the training dataset.

## Materials

### CTC cases

We constructed two independent CTC datasets for the experiments: a development dataset and a test dataset. The development dataset was constructed for providing samples of polyps for the training of 3D Glow for generating synthetic polyps and for providing samples of real polyps and normal anatomy for training of a 3D CNN for polyp detection. The independent test dataset was constructed for testing of the 3D CNN performance. The details of the training and testing of the polyp detection are explained in the section of “Polyp classification study” in “Evaluation methods” section.

We constructed a retrospective development dataset of 203 patients with 269 colonoscopy-confirmed polyps $\ge$ 6 mm in size from cases of various clinical CTC screening trials [19–22]. Most of the patients had been prepared with a cathartic bowel preparation and oral contrast (fecal tagging) by iodine and/or barium. The patients had been scanned in two (supine, prone and/or decubitus) positions by use of a total of 11 CT scanner models (Siemens, Philips, Toshiba, and GE Medical Systems) at 120 or 140 kVp, 0.54–0.97 mm pixel spacing, 0.75–5.00 mm slice thickness and 0.7–2.5 mm reconstruction interval. The spatial locations of the polyps on the CTC images were established by experienced radiologists.

We constructed an independent test dataset of 36 patients with 64 colonoscopy-confirmed polyps from two distinct subgroups of patients. The first subgroup of these patients (20 patients with 45 polyps) was prepared by use of a reduced cathartic bowel preparation with 18 g of magnesium citrate and fecal tagging by 50 ml of non-ionic iodine. The CTC acquisitions (SOMATOM Definition, Siemens Healthcare, Erlangen, Germany) were performed at 140 kVp with 64$\times$0.6 mm collimation, pitch of 0.85, rotation time of 0.5 s, 1.0 mm slice thickness and a 0.6–0.7 mm reconstruction interval. The second subgroup (16 patients with 19 polyps) was prepared by use of the previously established protocol of polyethylene glycol solution plus contrast medium [23]. The CTC acquisitions (Acquilion, Canon Medical Systems, Tochigi, Japan) were performed at 120 kVp with 16 or 64 $\times$ 0.6 mm collimation, pitch of 0.81–0.94, rotation time of 0.5 s, 0.5 or 1.0 mm slice thickness and 0.8 or 1.0 mm reconstruction intervals.

### Extraction of VOIs

For the experiments, we extracted three types of VOIs by use of the development dataset: 100 VOIs of real polyps, 100 VOIs of synthetic polyps and 100 VOIs of normal anatomy. The 100 VOIs of real polyps were those of randomly sampled real polyps. The VOIs of synthetic polyps were generated by 3D Glow after training with the VOIs of the 269 real polyps from the development dataset. The VOIs of normal anatomy were the VOIs of randomly sampled FP detections obtained at 100% polyp detection sensitivity by the 3D detection module of our previously developed CADe system [24–26].

**Fig. 5:**
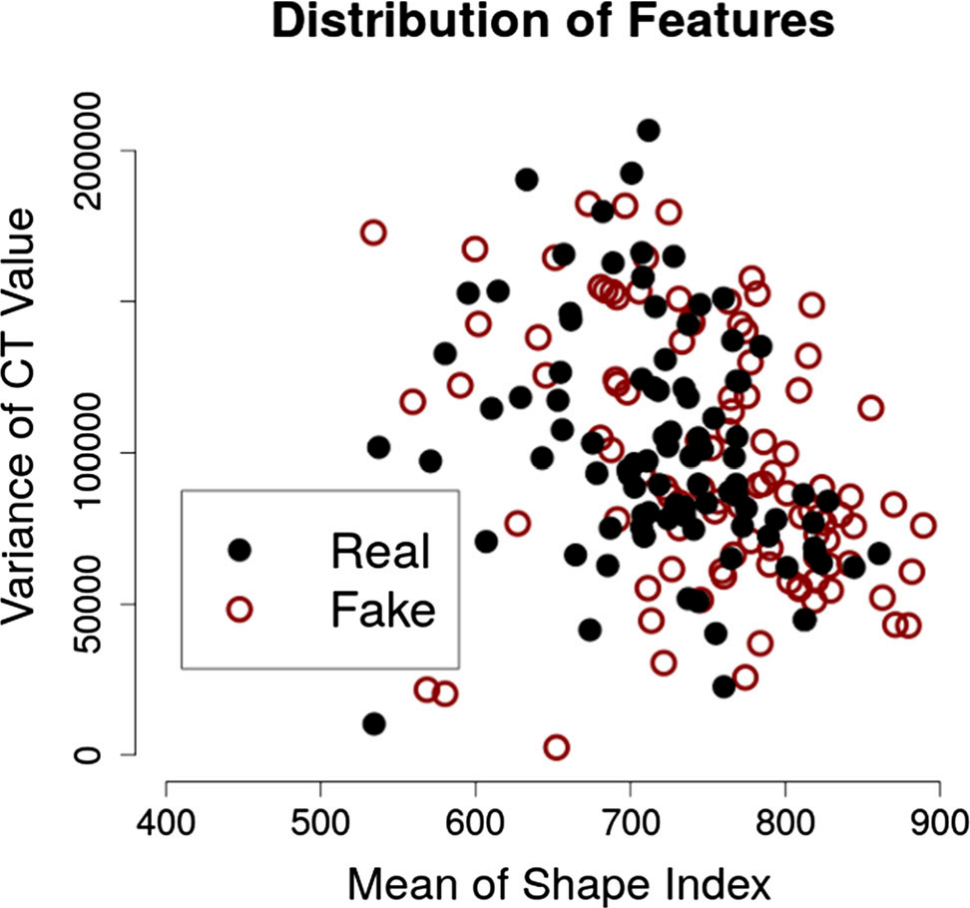
Visual comparison of the volumetric shape and texture features of the 100 real polyps (“Real”) and 100 3D Glow-generated synthetic polyps (“Fake”)

Figure 4 shows examples of the VOIs of the real polyps and 3D Glow-generated synthetic polyps that were included in our experiments. The synthetic polyps look visually indistinguishable from real polyps. Figure 5 shows a visual comparison of the radiomic values of the polyp regions of the real and synthetic polyps in terms of two radiomic features that we had previously identified as being most effective in the differentiation of true polyps from non-polyps in CADe [27]. The comparison indicates that the synthetic and real polyps are radiomically indistinguishable.

For independent testing, we acquired all of the VOIs of the polyp candidates that were detected from the test dataset (see the subsection of “CTC cases”) by the detection module of our previously developed CADe system. There were 92 true-positive (TP) detections $\ge$ 6 mm in their largest diameter (6–9 mm: 61 TPs) and 6519 FP detections. To calculate meaningful area-under-the-curve (AUC) values for receiver operating characteristic (ROC) analysis, we balanced the number of TP and FP detections in the test dataset by data augmentation of the TP VOIs with 3D rotations. This yielded a total of 12,407 VOIs for independent testing.

## Evaluation methods

### Observer study

Three experienced observers (two physicians and one physicist, each with experience of reading > 500 CTC studies) attempted to differentiate the VOIs of the 100 real polyps from those of the 100 3D Glow-generated synthetic polyps. The VOIs were loaded to a commercial CTC reading workstation (AZE Virtual Place Raijin, Canon Inc., Tokyo, Japan), where they were presented to the readers in random order. The readers were instructed to evaluate the VOIs interactively by use of the standard 2D and 3D CTC reading modes. For each VOI, the reader recorded his/her confidence that the VOI contained a real polyp, on a scale from 0 (synthetic polyp) to 10 (real polyp). The reading time was unlimited.

The discrimination performance was evaluated by use of ROC analysis, where the confidence levels recorded by each reader were analyzed by use of the pROC package (version 1.16.2) [28] in R (version 3.6.3) [29]. The ROC curves were generated by use of binomial fitting. The 95% confidence intervals were computed by use of bootstrapping with 1000 replicates. The difference of the AUC value from 50% (the level of not being able to tell the difference between a real polyp and a synthetic polyp) was tested by use of a bootstrap test with 1000 replicates, where $p<0.05$ indicated a statistically significant difference.

### Polyp classification study

We trained our previously developed 3D DenseNet CNN [30] to detect polyps with five data augmentation approaches: baseline augmentation, nonlinear augmentation, 3D GAN with baseline augmentation, 3D Glow with baseline augmentation and 3D Glow with nonlinear augmentation. The baseline augmentation included random flipping and/or 1–3-times zooming of the VOIs. The nonlinear augmentation included the baseline augmentation plus 3D shifting, 3D rotation and/or application of Gaussian noise to the CT values of the VOIs. The GAN-based augmentation was based on our previously developed 3D self-attention GAN method for generating synthetic 3D polyp VOIs [15].

For the training of the 3D DenseNet, 200 VOIs of real polyps and normal anatomy were extracted from the development dataset as described in section “Extraction of VOIs”. In the baseline and nonlinear augmentation approaches, the 3D DenseNet was trained with the 200 VOIs augmented by each approach. In the Glow- and GAN-based augmentations, the training dataset also included 100 synthetic polyp VOIs generated by each method.

After training with data augmentation, the 3D DenseNet was tested with the 12,407 VOIs extracted from the independent test dataset. The likelihoods for the presence of a polyp in a VOI as estimated by the 3D DenseNet were analyzed by use of the pROC package (version 1.16.2) [28] in R (version 3.6.3) [29]. The classification performance was evaluated for the clinically significant polyp size categories of $\ge$ 6 mm (all polyps) and 6–9 mm (small polyps) by use of the AUC as the performance metric. Bootstrapping with 1000 replicates was used for calculation of the 95% confidence intervals of the AUC values as well as for testing of the difference of the AUC values, where $p < 0.01$ indicated a statistically significant difference.

**Fig. 6:**
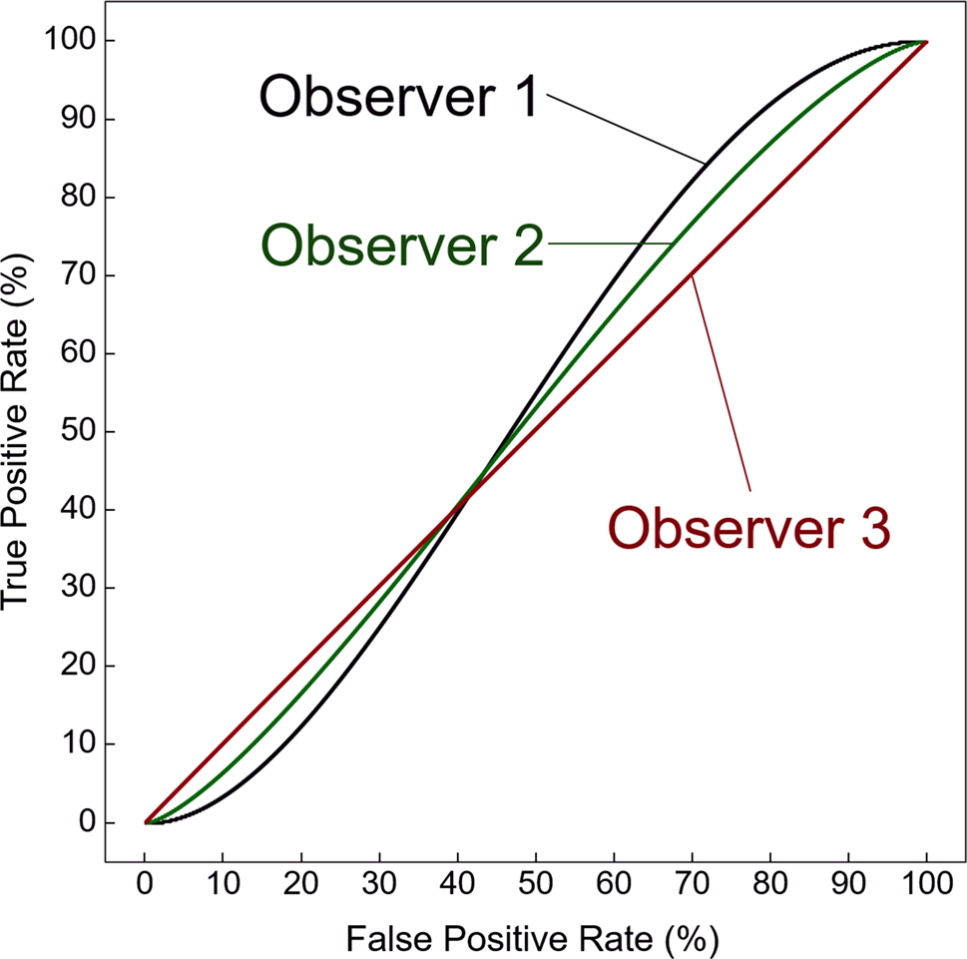
ROC curves of the three observers for the differentiation between synthetic polyps and real polyps

**Table 1:** The AUC values, 95% confidence intervals (CIs) and *p* values of the three ROC curves in Fig. 6

	AUC (%) (95% CI)	*p* value
Observer 1	52.7 [40.7, 62.5]	0.65
Observer 2	51.9 [43.6, 60.3]	0.81
Observer 3	50.3 [42.8, 59.1]	0.84

**Fig. 7:**
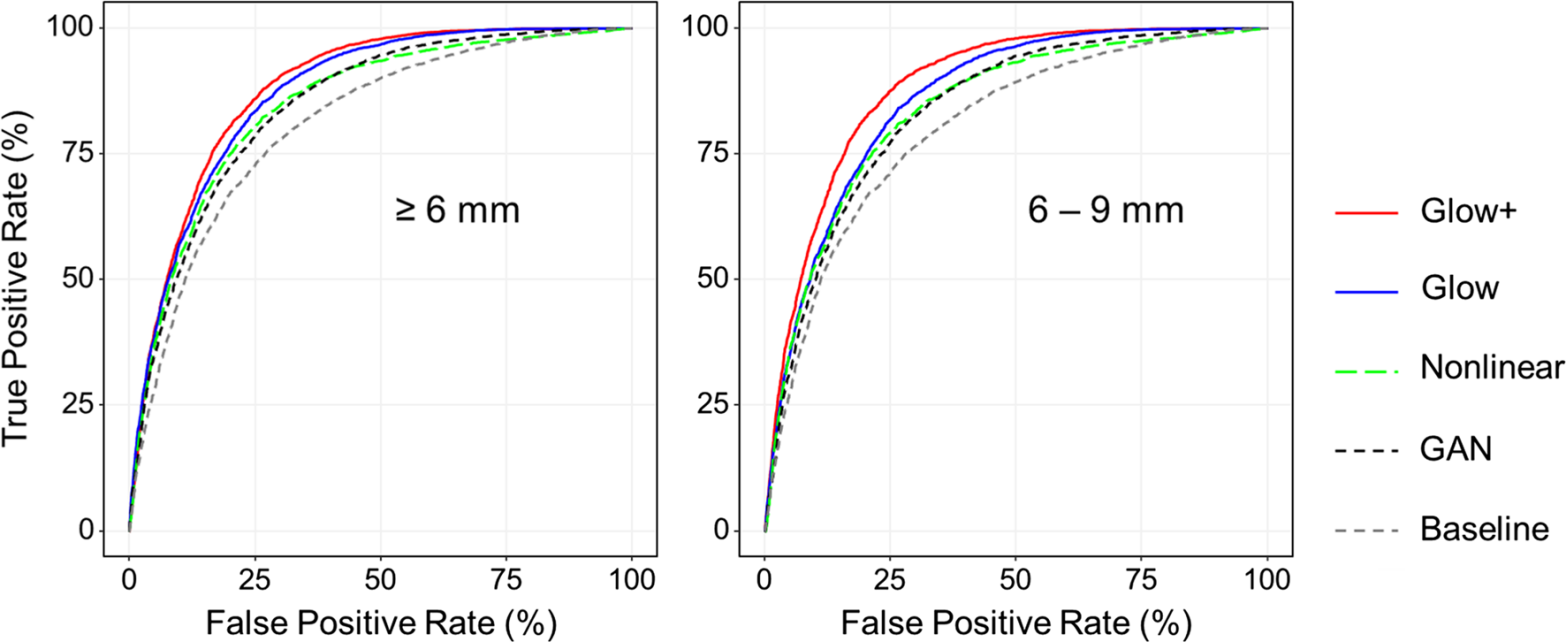
ROC curves representing the performance of the 3D DenseNet in the classification of polyp candidates with respect to the five different augmentation approaches used for the training step. The left plot shows the ROC curves for all polyps in our test dataset, whereas the right plot shows the ROC curves for clinically significant small polyps. *Abbreviations* Glow+ = 3D Glow with nonlinear augmentation; Glow = 3D Glow with baseline augmentation; Nonlinear = nonlinear augmentation only; Baseline = baseline augmentation only; GAN = 3D GAN with baseline augmentation

## Results

### The observer study

Figure 6 shows the ROC curves of the performance of the three observers in the differentiation of the 3D Glow-generated synthetic polyps from real polyps. The ROC curves indicate that the observers were unable to differentiate between the polyps. Table 1 shows an analysis of the AUC values of the ROC curves and their statistical significance with respect to difference from 50%. None of the AUC values of the ROC curves were statistically significantly different from 50% ($p \ge 0.65$).

### The polyp classification study

Figure 7 shows the ROC curves representing the classification performance of the 3D DenseNet between polyps and non-polyps based on the training with the different augmentation approaches. The ROC curves on the left show the classification performance for all polyps in our test dataset, whereas the ROC curves on the right show the performance for the clinically significant small polyps (6–9 mm in size), the detection of which is generally challenging. In both cases, the use of the 3D Glow-based augmentation approaches, Glow+ (red curves) and Glow (blue curves) yielded a higher performance than did any of the other augmentation approaches, and Glow+ yielded the highest performance.

**Table 2:** AUC values obtained from the ROC curves in Fig. 7, and their pairwise comparison results

	Glow+	Glow	Nonlinear	GAN	Baseline
$\ge$ 6 mm					
Glow+	88.1 [87.5, 88.7]	0.00013	< 0.0001	< 0.0001	< 0.0001
Glow	0.9	87.2 [86.5, 87.8]	< 0.0001	< 0.0001	< 0.0001
Nonlinear	3.4	2.5	84.7 [84.1, 85.4]	0.49	< 0.0001
GAN	3.5	2.6	0.1	84.6 [83.9, 85.3]	< 0.0001
Baseline	7.0	6.1	3.6	3.5	81.1 [80.3, 81.8]
6–9 mm					
Glow+	88.7 [88.1, 89.2]	< 0.0001	< 0.0001	< 0.0001	< 0.0001
Glow	2.6	86.1 [85.6, 86.7]	< 0.0001	< 0.0001	< 0.0001
Nonlinear	4.6	2.0	84.1 [83.4, 84.8]	0.51	< 0.0001
GAN	4.8	2.2	0.2	83.9 [83.3, 84.6]	< 0.0001
Baseline	8.3	5.7	3.7	3.5	80.4 [79.6, 81.4]

The diagonal components show the AUC values in percentage with the 95% confidence interval in square brackets. The upper triangle shows the *p* values obtained from a bootstrap test (1000 replicates) for the pairwise comparison of the AUC values. The lower triangle shows the pairwise differences in the AUC values

Glow+ = 3D Glow with nonlinear augmentation; glow = 3D Glow with baseline augmentation; nonlinear = nonlinear augmentation only; baseline = baseline augmentation only; GAN = 3D GAN with baseline augmentation

Table 2 lists the AUC values obtained from the ROC curves in Fig. 7, as well as their pairwise comparison results. The upper table shows the results for all polyps, whereas the lower table shows the results for the clinically significant small polyps (6–9 mm in size). In both cases, the 3D Glow-based augmentation approaches (Glow+ and Glow) yielded statistically significantly higher AUC values than did the other augmentation approaches, and Glow+ yielded the highest AUC values.

## Discussion

Although deep learning has made it relatively easy to obtain state-of-the-art results of various medical imaging tasks, in CTC, the available training datasets are relatively small, and the numbers of clinically significant polyps are even smaller. In this study, we investigated the feasibility of developing a flow-based generative model for simulating colorectal polyps to improve the training of 3D CNNs in CADe for CTC. The results of our experiments indicate that this is indeed feasible.

In the first experiment, we performed an observer study to compare the visually observed characteristics of 3D Glow-generated synthetic polyps with those of real polyps. To the best of our knowledge, this is the first study to show that it is possible to generate VOIs of synthetic polyps that are visually equivalent to VOIs of real polyps in CTC.

In the second experiment, we investigated the effect of the training of deep learning (3D DenseNet) with synthetic polyps generated by 3D Glow on the differentiation of polyps from non-polyps in CTC. To the best of our knowledge, this is the first study to show that data augmentation by synthetic polyps can be used to yield a statistically significant performance improvement in deep learning for CTC. We demonstrated that the use of synthetic polyps generated by 3D Glow outperformed the use of traditional nonlinear and GAN-based augmentations, and that the classification performance was highest when 3D Glow was used in combination with a nonlinear augmentation.

The observed improvement in the polyp classification performance by use of the 3D Glow was high for small polyps (6–9 mm in size). This result is important, because it is the detection of small polyps that remains challenging in CTC, whereas many clinical studies have demonstrated that existing CADe systems can detect large polyps in CTC at a high accuracy [31, 32]. Therefore, 3D Glow could have a meaningful impact in improving the detection of small polyps.

We performed the polyp detection by use of volumetric analysis of 3D CT colonography data, because this enables deep learning to review the complete region of the colon regardless of obstructions such as collapsed or poorly distended colon segments. Although other methods such as virtual endoscopic views have been used previously for polyp detection by deep learning [6], such approaches are limited to well-distended regions of the colon, whereas obstructed regions that can hide clinically significant polyps or masses would need to be identified and reviewed by other means. Also, the visual appearance of a virtual endoscopic view depends on the rendering algorithm and its visualization parameters, whereas 3D CT colonography data are readily standardized by Hounsfield units. Thus, the volumetric analysis provides the most uniform approach for the application of deep learning to polyp detection in CT colonography.

This study had several limitations. First, the number of datasets was necessarily relatively small. Second, there are various other data augmentation methods besides the nonlinear image transforms and the GAN-based method that we used as reference augmentation methods in this study. However, a thorough systematic assessment of their comparative performance against 3D Glow will require a separate study. Third, the study could be expanded by consideration of the unique demands of CTC, such as various bowel preparations or polyps that tend to be underrepresented in CTC datasets. Addressing such issues provides topics for future studies.

## Conclusion

We developed a 3D CNN based on a flow-based generative model for generating synthetic colorectal polyps in CTC. Our results indicate that the generated synthetic polyps are visually indistinguishable from real polyps and that data augmentation by such polyps can significantly improve the performance of deep learning in CADe for CTC.
